# Cheminformatics and the Semantic Web: adding value with linked data and enhanced provenance

**DOI:** 10.1002/wcms.1127

**Published:** 2013-01-08

**Authors:** Jeremy G Frey, Colin L Bird

**Affiliations:** Chemistry, Faculty of Natural Environmental Science, University of SouthamptonHighfield, Southampton, SO17 1BJ, UK

## Abstract

Cheminformatics is evolving from being a field of study associated primarily with drug discovery into a discipline that embraces the distribution, management, access, and sharing of chemical data. The relationship with the related subject of bioinformatics is becoming stronger and better defined, owing to the influence of Semantic Web technologies, which enable researchers to integrate heterogeneous sources of chemical, biochemical, biological, and medical information. These developments depend on a range of factors: the principles of chemical identifiers and their role in relationships between chemical and biological entities; the importance of preserving provenance and properly curated metadata; and an understanding of the contribution that the Semantic Web can make at all stages of the research lifecycle. The movements toward open access, open source, and open collaboration all contribute to progress toward the goals of integration.

## INTRODUCTION

Cheminformatics is usually defined in terms of the application of computer science and information technology to problems in the chemical sciences. Brown^1^ introduced the term *chemoinformatics* in 1998, in the context of drug discovery, although informatics techniques have been applied in chemistry since 1950s and cheminformatics now relates to a broader set of contexts. Willett,^2^ who uses the name ‘chemoinformatics’, provides a brief history of the development of the discipline. Warr,^3^ who parenthesizes the ‘o’ in the title of her article gives a more comprehensive description. We follow the Journal of Cheminformatics^4^ in adopting the shorter name. Both articles describe the application of cheminformatics to drug discovery and how the latter has influenced the development of cheminformatics. The allied discipline of bioinformatics evolved more recently, in response to the vast amount of data generated by molecular biology, applying mathematical, and computational techniques not only to the management of that data but also to understanding the biological processes, pathways, and interactions involved. In his paper about the commercialization of bioinformatics, Jones^5^ sums up the key factors that have influenced the development of the discipline. Sukumar et al.^6^ have reviewed the interaction between cheminformatics and bioinformatics. They identify data transformation and data fusion as vital aspects on which further integration depends, noting the importance of semantics for achieving a more holistic approach. The goal is to establish systems chemical biology as a discipline, as outlined by Oprea et al.^7^ Very recently, Wild et al.^8^ have surveyed the current status of systems chemical biology, particularly with regard to the Semantic Web. Chepelev and Dumontier^9^ refer to the emergence of systems chemistry, suggesting the development of a more systematic view of chemical experiments in an interdisciplinary context. However, they do not include among their references the 2008 review of systems chemistry by Ludlow and Otto,^10^ which considers this emerging discipline from a complex systems perspective. They restrict themselves to synthetic systems in solution, for example, combinatorial chemistry, but also cover other multivariate systems, including models that might contribute to the understanding of biological systems.

With increases in computing power came not only a growth in capability but also a dramatic expansion of the volume of data produced and a demand for more sophisticated information technology to keep pace with the increased quantities of data. As chemistry and biology evolved, the greater information processing capacity stimulated differentiation and specialization within these disciplines, leading to subcategories within each field. At its most basic, chemometrics applies mathematical and statistical methods to the design of experiments with chemical systems, the analysis of the data obtained, and the understanding of those systems. As such, chemometrics clearly predates cheminformatics. Similarly, biostatistics, the application of statistical methods to biology, came before bioinformatics.

**Figure 1:**
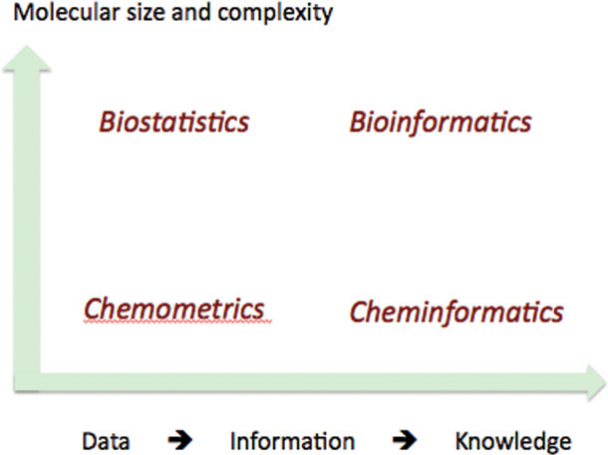
The related and complementary disciplines of Bio/Chemo statistics and informatics

In general terms, chemometrics does not entail knowledge of chemical structure, being concerned mainly with obtaining information from data. The same might be said of biostatistics. Cheminformatics and bioinformatics seek to discern the patterns in the information, to elicit chemical and biological knowledge. Any distinction between these two branches of informatics relies mainly on the size and complexity of the molecules studied. Figure 1 shows the relationship between the four disciplines, but without clear divisions owing to the potential overlaps. The two informatics disciplines take their respective sciences, distinguished here by the size and complexity of the molecules studied, further along the data–information–knowledge sequence. The scope for applying all four remains large, as demonstrated in the recent review of the enumeration of chemical space by Reymond et al.^11^

Cheminformatics also embraces the distribution, management, access, and sharing of chemical data, and it is to these aspects of the discipline that the Semantic Web has so much to offer, by integrating heterogeneous sources of chemical, biochemical, biological, and medical information. The twenty-first-century e-Science and e-Research programs stimulated progress toward a more holistic and data-centric approach to the chemical sciences: Kim^12^ recognized the importance of cyberinfrastructure in his editorial for the 2006 focus issue of the Journal of Chemical Information and Modelling. In his 2009 overview of Semantic Chemistry, Adams^13^ describes chemistry as a ‘conservative discipline’, having noted its comparative reluctance to evolve a culture of data and knowledge sharing, but adds that chemistry is now participating in the Semantic Web.

Hawizy^14^ discusses a ‘semantification workflow’ for exploiting the potential of linked data, which she argues will have a profound impact on the development of science in the twenty-first century. However, she acknowledges the inhibitors to accessing chemical information sources. Frey^15^ discusses the significance of the support of virtual organizations and the need for the coordinated development of ontologies for chemistry, and other nonbiological disciplines. A Semantic Science blog makes a plea that we do not forget the data from small projects, which can become big data when aggregated.^16^ Semantic Web technologies can achieve that aim, even though the social and commercial aspects of using the Semantic Web remain areas in need of work. The linkage of data and resources is a recurrent theme in ‘The Fourth Paradigm’, a book about data-intensive scientific computing.^17^ With regard to chemistry, Frey^15^ stresses the importance of links between laboratory records and the computer systems that hold the data, but notes the need for better ways to maintain those links. Later in the same article, he says: ‘It is the links that add value; but getting people to add them, or add sufficient information that they can be created automatically, is proving to be hard.’ Links can reduce the time to data discovery, but the provenance of that data, and indeed of computational services, remains a concern. The outputs of one phase of the research lifecycle are often inputs to another phase: semantic links can help to ensure that the provenance trail remains intact. The so-called ‘Dukes University scandal’ strongly endorses this point. Although not directly related to chemistry, the article by Ince^18^ amply demonstrates the importance of provenance information for both audit and reproducibility. However, to reinforce the need to capture the relevant metadata, researchers must perceive advantages in terms of, for example, improved accuracy, easy record keeping, and less repetition^15^: the ultimate aim is Curation@Source.^19^ This review shows how the Semantic Web is beginning to have an impact on cheminformatics by aiding the discovery and reliable reuse of data, facilitating automated processing of that data, as well as providing enhanced provenance.

We start our discussion by considering the generation of chemical data and the nature of this data in comparison to other related disciplines. This data needs to be managed, an increasing difficult task given the quantities of data now available. To be useful the data needs to be integrated, abstracted, and made discoverable and deliverable in an intelligent and intelligible manner to other chemists and researchers in general. We discuss the value of chemical identifiers, metadata, vocabularies, linked data, provenance, and how these are being achieved with Semantic Web technologies and ontologies. We return to an overview of the application of these ideas to the overall research lifecycle to place them more fully in context, to then talk about the deployment of the Semantic Web, workflows, open data, and, more generally, interoperability and semantically enhanced provenance.

## DATA MATTERS

Chemists have always generated data, and the chemical sciences have relied on data to advance the understanding of the discipline. Vast quantities of experimental data are now available, owing to new spectroscopic and visualization techniques, combinatorial and high-throughput methodologies, and increasingly complex computational investigations: quantum mechanical structural determinations and simulation dynamics. Each year computing facilities become more powerful, and indeed have to do so, just to keep pace with the expanding volume of data. The imperative to make the best possible use of the data available, especially given the costs associated with its collection, raises issues with preservation, curation, discovery, and access. These issues are at the core of the Semantic Web vision.^20^,^21^ Handling this data and extracting information and knowledge from it almost becomes a discipline in its own right, the science of informatics.

Informatics depends on data, but it is essential that data is reliable, and of an assured quality; moreover, that quality must be capable of being assessed. This requirement is particularly pertinent to the drug discovery process, for which the emphasis of cheminformatics has shifted from techniques to the management, curation, and integration of the large amounts of potentially useful data, with increasing dependence on Web services (see Ref 22 and references therein). Drug discovery has evolved from being an essentially empirical process through rational design and large-scale, high-throughput experiments to approaches based on genomics, which generate large amounts of potentially useful data.^23^ Drug discovery also relies on bioinformatics. Curcin,^24^ reviewing Web services in the life sciences, acknowledges the potential importance of Semantic Web technologies, but remarks that a systematic and standardized approach is needed. Tetko^25^ compares the adoption of Web services by the bioinformatics and cheminformatics communities, stressing that the differences arise from the quantity of data involved and the scale of public funding to the bioinformatics area. The complexity of ownership, perceived potential to generate income, on top of the native complexity and scale inherent in the descriptions of chemistry (chemical space) lead to fundamental problems in the management of the data. It is essential to address these problems if data intensive chemistry is to realize its potential for integrating with other material and life science disciplines that are underpinned by chemistry.

## BOX 1: WEB SERVICES

In the early days of scientific computing, researchers wrote their own, almost inevitably bespoke, code. Subsequently, application packages and software libraries were developed, enabling considerable efficiency gains. The next key evolutionary step was the service-oriented architecture (SOA) approach, with the sharing of functionality increasingly provided through Web-based resources. A measure of the extent of the services available in the bioinformatics area is provided by the BioCatalogue,^26^ which maintains a list of these services and service providers.

Web services can be used for functions ranging from information retrieval to performing calculations. These services offer well-defined programming interfaces that are essentially independent of the programming languages and platforms used to access them. The formal definitions of Web services interfaces, such as the WSDL^27^ and SOAP^28^ specifications, are beyond the scope of this review. However, the simpler REST (Representational State Transfer) architecture is now the preferred approach to implementing Web services,^29^ a choice that presumably also influences the design of Web services deployed in drug discovery. Another design consideration is that of thin versus thick clients.^30^ Thick clients employ a formal, machine-processable, interface definition, whereas thin clients rely on the server to interpret each request. Enterprise applications require rigorous specifications of business requirements, so prefer thick clients.

## Data Management and Integration

Frey notes a preference among laboratory scientists for storing data in flat files (in computers hidden under desks), which is not a good approach for curation, reuse, or preservation.^15^ He examines alternative for larger-scale preservation, such as relational database and laboratory information management systems (LIMS), and discerns a need to cover ‘the middle ground between the uncontrolled flat files and the rigid relational database’. Reese^31^ suggests that relational databases are appropriate for data that changes frequently and for which maintaining integrity is important. He argues that data that does not change is best preserved in flat files, in tabular form wherever possible, and also proposes that, as well as the raw data, the archive should also contain a *codebook* that records how the data is entered and the descriptive metadata.^31^ The Semantic Web is also capable of covering the middle ground and capturing the same information, given sufficient attention to metadata descriptions. In recent years, storage and computation ‘in the cloud’ have added a fresh dimension to the management of large volumes of data. Several of the references cited in this review mention cloud computing, but none cover it as a specific topic.

On a smaller scale, Alsberg and Clare^32^ have used a wiki in conjunction with version control software to manage the data objects generated by their chemometric research projects, enabling them to integrate project information with data. They point to the advantages of flexibility and communication, but acknowledge a number of shortcomings, some of which are the undesirable consequences of flexibility. From the perspective of this review, the lack of semantic annotation is significant: the data is not curated for machine processing.

In 2006, Taylor^33^ reviewed the use of electronic laboratory notebooks (ELNs). His focus was on commercial systems and the regulatory considerations for electronic laboratory records, remarking that academic researchers had shown little interest in ELNs. The two exceptions he noted were the CombeChem^34^ and SmartTea^35^ projects, to be discussed more fully in later sections of this review.

Considering the volume and complexity of the data available for pharmaceutical R&D, Slater et al.^36^ argue that it is not enough to bring together data and information from multiple sources. Semantics are necessary to interpret the information and derive knowledge. They propose a knowledge representation scheme that corresponds to the Semantic Web vision of data and resources described for use by humans and machines. In 2009, Wild^37^ reviewed the use of data mining, together with Semantic Web techniques, for achieving the semantics-based integration envisioned by Slater et al.^36^ The following year, Guha et al.^38^ reviewed advances in the data mining of large heterogeneous chemical datasets, noting throughout the influence of semantic technologies on infrastructures for processing chemical information. Stephens et al.^39^ have used an RDF (Resource Description Framework) data model to aggregate the disparate data used for drug discovery.^40^ McCusker et al.^41^ have created a data warehouse based on Semantic Web technologies, as a tool for the caGrid developed by the US National Cancer Institute (NCI). The Chem2Bio2RDF project illustrates what can be achieved by using semantics to integrate data from multiple chemical and biological sources.^42^ Chem2Bio2RDF demonstrates how the federation of resources can facilitate search.

The RDF data model describes entities in terms of subject–predicate–object expressions, commonly known as triples. These expressions are held in a *triple store*, which is a database optimized for the storage and retrieval of triples.^43^ Frey^44^ describes the choice of RDF for the CombeChem project, and considers the implications of using RDF.

Hastings et al.^45^ assert that the application of cheminformatics is critically dependent on the data exchange process, and are developing the Chemical Information Ontology (CHEMINF) to facilitate the precise description of chemical entities. Their motivation is twofold: (1) to provide a common reference point for interrelating terminology developed independently; and (2) to enable Semantic Web tools to integrate data from disparate sources for reuse in data-driven research. They state their aim to be the adoption of CHEMINF as a standard by the cheminformatics community.

Two of the coauthors of the CHEMINF paper, Chepelev and Dumontier,^9^ report related activities intended to improve the ability of Semantic Web tools to federate chemical data and information. SADI (Semantic Automated Discovery and Integration) is a framework that deploys RESTful Semantic Web Services. The novel feature is that SADI services generate an output class by annotating the input class, thus preserving the provenance of the service explicitly. They also implement CHESS (Chemical Entity Semantic Specification) for representing chemical entities and their descriptors.^46^ A key aim for CHESS is to enable the integration of data derived from various sources, thereby facilitating better use of Semantic Web methodologies.

The integration and aggregation of data from multiple sources reaches a zenith in drug discovery research. Blomberg et al.^47^ consider a range of initiatives aimed at increasing the interoperability of data and information, paying particular attention to semantic approaches and the use of Semantic Web technologies. They describe the formation and objectives of the Open PHACTS consortium, which will adopt a Semantic Web approach to address the bottlenecks in small molecule drug discovery.

## Discovery and Access

Discovery techniques that exploit the semantics of document content were in use well before the Semantic Web concept emerged. Jiao and Wild^48^ have applied text-mining techniques to biomedical literature, identifying characteristic data that enables them to extract information about chemical interactions. The SPECTRa-T project has used text-mining tools to extract chemical objects from electronic theses.^49^ A key difference is that SPECTRa-T stores the extraction results as RDF triples, allowing subsequent reuse and analysis with Semantic Web tools. Correspondingly, raw data, if sufficiently well described, should be susceptible to data mining techniques.

A recent example of the application of such techniques is the Collaborative Chemistry Database Tool (CCDBT),^50^ which is a repository for the raw data generated by computational chemistry packages. The authors recognize the vital importance of extracting metadata from the raw data, thereby enabling other computational chemists to reuse the data and/or the results derived. A sequence of parsers extracts metadata from the raw data and populates a database for subsequent query based on the metadata model.

However, text mining is retrospective discovery. Frey^15^ argues for a prospective approach to discovery, advocating the use of systems compatible with the Semantic Web in the laboratory, thus facilitating at source any subsequent discovery process. He warns, however, ‘it is crucial to appreciate that the researcher's view of the content of an information system can be, and usually is, quite different from the “view” required by a computer system attempting to act for, or with, that human.’ Both with retrospective or prospective approaches to gathering machine readable and processable data, the metadata is essential, and it is in handling this aspect that Semantic Web technologies come to the fore.

Taylor et al.^51^ demonstrate how Semantic Web technologies can be deployed in the storage and access of molecular structures and properties. Using unique identifiers and relationships, represented as RDF triples, they create a semantic database with the potential to enrich the exploitation of the data therein. One aspect of structure searching that has yet to feel the influence of the Semantic Web is that of finding chemical structures in patents, an area recently reviewed by Downs and Barnard.^52^

Frey^15^ also draws attention to the need for access control, in particular to protect intellectual property rights. He suggests that security models need to be rich but not overwhelming. Park has considered the requirements for secure collaborative work on the Semantic Web, including the need for efficient access control.^53^ The issues that arise are clearly generic and not confined to any specific application areas.

## DESCRIBING CHEMICAL DATA

A key and essential part of making data available via the Semantic Web is the existence of unique identifiers. In this requirement, the Semantic Web lines up with a considerable volume of work on chemical nomenclature as a way to create systematic (if not always unique) identifiers. Identifiers are the keys to the description of chemical structures and data although, of necessity, chemical identifiers should relate uniquely to a single structure. The chemical names used in publications are unique, but are not suitable for machine manipulation. Historically, the Wiswesser Line Notation^54^ gave way to SMILES (Simplified Molecular-Input Line-Entry Specification).^55^ Owing to some limitations with SMILES representations, IUPAC introduced the International Chemical Identifier (InChI) and its derivative, the InChIKey, which is a fixed-length hash code representation of the InChI itself.^56^ With the notable exception of polymers, the great majority of compounds, including organometallics, can be represented with InChI identifiers.

Williams^57^ notes the importance of the InChI for the Semantic Web in chemistry. Taylor et al.^51^ highlight the unique nature of the InChI and consider the construction of a uniform resource identifier (URI) from an InChIKey. Such URIs enable links between chemical properties, data, and publications, or entries in an ELN. Coles et al.^58^ have investigated the potential of the InChI for chemical information retrieval. Using the InChI strings for a corpus of 104 molecules whose crystal structures were published under the eCrystals/eBank project, they obtained high values for both precision and recall. Tests with other corpora were similarly encouraging.

Bhat^59^ discusses some potential difficulties with integrating the information needed for AIDS research and proposes methods and procedures to prepare data for a Chemical Semantic Web. He identifies as a specific challenge the unique naming of each substructure of a given compound and aims to build an ontology for the formal description of these components. Describing the relationships between chemical and biological entities can be of equal importance, especially for drug discovery. Guha et al.^38^ suggest that the aim should be a holistic view of the relationships between small molecules and biological systems. Although Williams praises the quality of the chemical information provided by Wikipedia,^57^ he points out that such descriptions are not machine-readable. However, DBpedia Live specifically aims to extract structured information from Wikipedia and convert it to RDF.^60^ Kohler,^61^ reviewing the three-volume set ‘Chemical Biology: From Small Molecules to Systems Biology and Drug Design’, emphasizes the importance of integrating chemical and systems biology.^62^ Describing the relationships between small molecules and biological entities will be key to that integration. The Semantic Web offers a formal mechanism for representing those relationships. For example, the ChEBI ontology^63^ captures the role of a chemical entity in a biological context. PubChem^64^ provides full descriptions of an extensive range of molecules, a chemical identifier (that is not unique in that while a PubChem identifier points to only one molecule many molecules have more than one PubChem identifier) with associated Web services, but does not include the semantic descriptions needed for machine reasoning.

### Metadata

Discussing the gap between bioinformatics and cheminformatics that existed in 2005, Curcin et al.^24^ identify the lack of integration with differences in databases and tools and a shortage of cross-domain expertise, but do not highlight the importance of metadata, which now plays a vital role in achieving interoperation between these disciplines. Metadata is crucial for realizing the vision of the Semantic Web and enabling machines to perform the essential steps of integration: discovering data, interrelating data, and initiating cheminformatics tasks that act upon that data.

The commonly cited description of metadata as ‘data about data’ runs into difficulties even in basic situations. Pancerella et al.^65^ give the example of a chemical formula, which can be metadata itself or be the object of other metadata, pointing out that the ‘about’ view can depend on perspective. Metadata is at the heart of their collaboratory for the multiscale chemical sciences (CMCS). They attach particular importance not only to discovering data across scales but also to preserving its provenance, goals that nearly 10 years later are regarded as essential. Moreover, the concerns they expressed about enforcing metadata standards across communities are in many ways alleviated by the tools of the Semantic Web, which provide, and work with, semantic metadata.

The formal recording of semantic metadata relies on ontologies, which are discussed in a later section. Ontology development is a rapidly evolving area and there has been a tendency for each group to create an ontology that meets its own needs. Although a set of standard chemical ontologies might seem desirable, the concern about alienation expressed by Pancerella et al.^65^ remains pertinent. Fortunately, infrastructures based on RDF, for example, do permit interoperation. The reuse of parts of existing ontologies is becoming more common and systems are becoming available for recording metadata, for example, the Investigation/Study/Assay (ISA) infrastructure.^66^ ISA assists with the reporting of experimental data, using community-agreed minimum metadata descriptions, thus ensuring that the metadata is sufficient to provide confidence in the data.

The reliability of metadata depends strongly on its capture as early as possible in the research lifecycle. Frey^19^ makes a strong case for designing curation into research practices, which would require metadata to be captured in context, as the data itself is generated. Capture at source requires a combination of manual and automatic recording: for manual recording, it is essential that recording is easy and, insofar as is possible, places no additional burdens on researchers; automatic data acquisition should capture context as well as data. Frey^34^ provides several examples of projects that have tackled the issues of curation, notably CombeChem. However, with regard to automatic data capture from networked instruments, Frey^15^ also sounds a cautionary note. There are still issues with regard to ensuring that the data produced by such instruments conforms to international standards and has high quality metadata in a form that is usable by Semantic Web technologies. In an editorial for Drug Discovery Today, Williams and Ekins^67^ express more general concern about the quality of much of the structure-based chemical data in the public domain, and make a case for government funding to support data curation. Previously, Williams^68^ had emphasized the similar need for careful curation to ensure data quality in his review of Public Compound Databases. In former times, this was the role of national standards organizations and the international professional scientific bodies (ICSU, IUPAC, IUPAP, etc.), but funding has not been available to keep pace with the validation needs of the growing data volumes.

### Vocabularies

A common vocabulary is fundamental to understanding and communication in cheminformatics and the Semantic Web, just as it is in most other spheres of human activity. Bhat^59^ sees the development of common vocabularies and general ontologies, amongst other technologies, as research directions for the chemical Semantic Web. However, for a vocabulary to be *common*, the terms it contains must be agreed and workable in practice. Moreover, the vocabulary must be in a form that is readable by Semantic Web tools. Frey^15^ notes that the capture of semantic relationships can lead to tension between freedom and control, in that controlled vocabularies inhibit the free text annotation with which researchers often feel more comfortable.

Many cheminformatics tools depend on metadata constructs that provide formal data descriptions by means of controlled vocabularies. Prominent among such constructs is the Chemical Markup Language (CML) for describing molecular species, first proposed in 1995. Since then, Murray-Rust and Rzepa^69^ have defined an XML Schema compliant form of CML. In 2011, Murray-Rust et al.^70^ described the semantics of CML, its conventions and dictionaries. Ref 71 contains a comprehensive list of CML publications, together with specifications and other information.

### Linked Data

Linked data, although generically an established concept, is fundamental to the Semantic Web. Tim Berners-Lee^72^ has published a range of notes concerning Web design issues, including four principles for putting linked data on the Web. The InChI and InChiKey, discussed in an earlier section, are very important for linking both raw and processed data that relates to molecules. The eCrystals archive^73^ uses InChI identifiers for linking to the data resulting from a single crystal X-ray structure determination, produced, for example, by the UK National Crystallography Service (NCS).^74^ The significant aspect of this service (both the NCS and eCrystals) is its preservation of links to all the raw and processed data, thus exposing the details of the structure refinement to scrutiny. This approach is not only interesting and useful but also provides a good exemplar for provenance conservation and a route to unconventional dissemination with accepted provenance.

To enable either a human user or a software agent to access linked data, URIs must be dereferenceable, by one of the variations described by Berners-Lee.^72^ The number and range of compliant datasets is growing, as shown by the W3C page that lists sources with dereferenceable URIs,^75^ describing them as ‘part of the emerging Web of Linked Data’. However, a search for the stem ‘chem’ produces only two matches, suggesting that the Semantic Web has much further to emerge if cheminformatics is to benefit from linked data. Curiously, the Linking Open Drug Data (LODD) Web site^76^ does not appear in the list of sources, despite being under the auspices of the W3C. The LODD Web site lists several interesting resources, available in a number of formats including RDF, and Samwald et al.^77^ describe the work of the LODD task force. They note that some of the LODD datasets are not fully open, owing to considerations that the task force is actively exploring (e.g., patient confidentiality).

ChemCloud^78^ adopts the linked data initiative in providing an infrastructure to integrate a range of chemical, biochemical, and pharmaceutical databases. This project recognizes that the formats in these sources present a challenge to semantic integration. Given the prevalent use of XML formats in these databases, ChemCloud has developed tools for converting the XML data to RDF.

In 2004, Murray-Rust and Rzepa^79^ published an article challenging the transclusion model on integrity grounds. They admit that their message is ‘slightly tongue-in-cheek’ but go on to propose a *datument* model, in which publications contain all the relevant parts, incorporated as the datument is published. Berners-Lee published his principles of linked data two years later, but it is perhaps notable that a search of all his design issues produces no matches for the stem ‘integr’ (to cover variants of ‘integrity’). Although capturing links is likely to remain a challenge in the context of chemical experiments, it is perhaps fortunate that ensuring that laboratory data is linked to some at least of its related information should suffice to prevent that data becoming isolated.

## PROVENANCE

Enhancing the mechanisms for recording and storing provenance is possibly an understated goal of the union of cheminformatics and the Semantic Web. Borkum et al.,^80^ describing the oreChem project, point out the importance of the relationship between the level of trust in reported results and the provenance, or pedigree, of the data from which those results were derived. Their words echo the earlier observations of Pancerella et al.,^65^ regarding the importance of provenance for the accuracy and currency of scientific data. To ease the checking of provenance and validity, repositories need as much information as possible about the data they contain, and Semantic Web technologies offer the means for capturing and preserving that information.

In 2005, Simmhan et al.^81^ published a survey of data provenance in e-Science. Although the CMCS is the only chemistry project they examine, they raise several general issues that remain pertinent today, including, but not limited to: rich provenance information can become larger than the data it describes, provenance usability depends on federating descriptive information, coping with missing or deleted data requires further consideration.

To some extent, these issues can be addressed by the use of inference techniques, which is a natural step, given the enabling technologies of the Semantic Web. Provenance Explorer generates graphical views of scientific data provenance by using rule-based methods to infer provenance relationships automatically.^82^,^83^ The system comprises a knowledge base of Web Ontology Language (OWL) files with relationships defined in the Semantic Web Rule Language (SWRL), an inference engine (Algernon), and a provenance visualizer.

The CombeChem project is an exemplar for capturing provenance information at source.^34^,^51^,^84^ This project also recognized the need for the descriptive information to be pervasive, for example, including units. The ChemAxiom set of ontologies includes *ChemAxiomMeta*, which is intended to allow the provenance of data to be specified.^85^

The need for provenance information to be reliable has potential significance for drug discovery, when molecular properties are computed: the provenance should show clearly the method of performing calculations. The Blue Obelisk Movement makes a similar point in the general cheminformatics context.^86^ Its members urge that chemical computations should satisfy the scientific tenet of reproducibility, but note the surprising difficulty of ensuring the reproducibility of a calculation. They go on to argue that a global chemical Semantic Web will be difficult to implement without the processes necessary for validating resources and methods. Hastings et al.^45^ also consider the provenance of calculated data to be particularly important, and use their Chemical Information Ontology (CHEMINF) to capture that information, for example, the parameters and the version of the code used to compute chemical properties.

## SEMANTIC WEB TECHNOLOGY

Maximizing the value of the Semantic Web to cheminformatics depends in part on the availability of good tools. Murray-Rust et al.,^87^ in a perspective article, published in 2004 and entitled ‘Representation and use of Chemistry in the Global Electronic Age’, discuss the importance of appropriate tools for all aspects of the Chemical Semantic Web. A 2006 survey of the technologies comprising the Semantic Web and its architecture provides a comprehensive set of references.^88^ This survey acknowledges the wide range of application areas without mentioning any specifically. Two years later, a survey of semantic e-Science applications describes chemistry as a ‘hot field’.^89^ The authors look forward to a promising future but note among the challenges two that remain pertinent today: existing data and social issues. Of the former, they say: ‘providing structured data already existing in legacy database according to an agreed ontology can be a very labor-intensive task’. The social issues relate essentially to willingness to contribute to the creation of the Semantic Web.

In their book *Introduction to Pharmaceutical Bioinformatics*, Wikberg et al.^90^ include a chapter about the Semantic Web that describes the standards and technologies in the context of cheminformatics and bioinformatics. Of all the Semantic Web technologies, arguably the most significant in terms of dependencies is RDF, the Resource Description Framework. In 2010, the Journal of Cheminformatics devoted a Thematic Series to ‘RDF technologies in chemistry’.^91^ Two of the papers in this series, about SADI^9^ and Chess^46^ have been covered in *Data Management and Integration*; the article by Samwald et al.^77^ about LODD has been covered in *Linked Data*. Another article in the series, by Willighagen and Brändle,^92^ addresses the use of RDF in chemistry specifically. The authors are generally optimistic about the future value of RDF technologies for chemistry, although they do question the usefulness of RDF for data in tabular forms and also sound a cautionary note about the inability of RDF to provide guarantees about data quality or data availability, for example.

Adams^13^ published an overview in 2009 that considered semantic markup languages for chemistry, such as CML, as well as Semantic Web technologies. Notably, he raises issues similar to those discussed by Chen et al.^89^ in 2006: the processing of existing data, which Adams refers to as ‘semantification’; and the sociocultural challenges. He observes that chemistry has lagged behind other disciplines in evolving a culture of data and knowledge sharing. As Frey^34^ noted when describing the CombeChem project: ‘All progress depends on individual scientists building on the results already produced by others’. Adams warns of the risk to progress in the biosciences in particular if chemistry continues to be reluctant to share its data.

The SPECTRa-T project has demonstrated the use of text-mining tools to extract semantic information from theses stored in legacy document formats, generating an RDF representation of the chemically relevant content.^49^ It is self-evident that the issues related to data extraction and sharing would be mitigated by publishing open access data together with the article to which the data relates, as advocated by Bachrach.^93^ This is an interesting development on a scheme that he and colleagues proposed a decade earlier, for journal articles to be marked up for reuse by readers.^94^ Bachrach suggests the use of Web 2.0 tools to assist with peer review in an open environment. Fox et al.^95^ envisage a wider use for Web 2.0 technologies, including SOAs for cheminformatics.

Storage and retrieval tools are essential, with an extensive range of triplestore implementations providing databases for persisting Semantic Web relationships, which consist of subject–predicate–object triples. The W3C standard for retrieving triples is SPARQL (SPARQL Protocol and RDF Query Language).^96^ Willighagen and Brändle^92^ discuss the use of SPARQL in cheminformatics, as do Chen et al.,^42^ when describing the Chem2Bio2RDF framework: these are just two examples.

SemanticEye is a system intended to improve the accessibility of electronic publications and associated data,^97^ along similar lines to those discussed above. The architecture of SemanticEye is based on the digital music model and relies on descriptive metadata that its stores as RDF. The original implementation used the Sesame framework^71^; subsequently, Casher and Rzepa^98^ have integrated SemanticEye with SPARQL.

### Ontologies

Ontologies for chemistry are not yet as well developed as those in the life sciences, but several initiatives are making encouraging progress. The first Casher and Rzepa^97^ paper describes SemanticEye as an ontology with associated tools. Other groups have also created formal semantic descriptions as taxonomies and ontologies, in many cases to meet their own needs. The ChemCloud initiative is, to some extent, an attempt to contain this proliferation, but it still requires new ontologies to represent the information in existing databases.^78^ Currently, ChEBI (Chemical Entities of Biological Interest)^63^ is the most established ontology in chemistry, as described by Adams et al.^99^ with a subsequent update by de Matos et al.^100^ Adams^85^ is also one of the originators of the ChemAxiom set of ontologies, which aims to provide a framework for the formal description of chemistry, in the form of a set of interoperable ontologies that describe both chemical concepts and chemical data.

The CHEMINF ontology, as described in *Data Management and Integration*, is particularly concerned to cater for the exchange of data about chemical entities with biological and bioinformatics applications.^45^ As covered fully in the paper, CHEMINF extends several ontologies that are important in the biological context. Although the authors acknowledge the influence of CombeChem^34^ they do not refer to the development of ChemAxiom,^85^ possibly owing to concerns about the ChemAxiom approach, for example, that it does not provide dereferenceable URIs. All three are domain-specific ontologies that aspire to integrate with upper ontologies, particularly those in the Open Biomedical Ontologies (OBO) format.^101^ CHEMINF also provides mappings to the Blue Obelisk Descriptor Ontology (BODO), which is covered in the 2011 review of the Blue Obelisk movement five years after its inception.^102^

Choi et al.^103^ have generated a small molecule ontology (SMO) to address the problem of integrating the properties of small molecules with data relating to biological activity. They emphasize the importance of Semantic Web technologies for both the development and exploitation of their SMO. On a broader level, Chen and Xie^104^ have surveyed the use of Web ontologies in drug discovery, which is an activity that manifestly depends on the integration of chemical and biological data. One rather specific example of the use of ontologies in this respect is the semantic mining of patents.^105^

Under the auspices of the CombeChem project, Frey et al.^35^ adopted a human computer interaction (HCI) approach to designing an information system for capturing the data and metadata recorded by chemists during an experiment. From a *Smart Lab* perspective, CombeChem used RDF to classify chemical descriptors and demonstrated the explicit capture of the provenance of an experiment.^34^ The Smart Tea project developed an ontology to model the Materials and Processes comprising the experiment, as one part of a system to support the experimental process from planning through to publication (at source). Representations of experiments at both the planning and enactment stage are at the core of the oreChem infrastructure: the model enables researchers to describe both the prospective and retrospective provenance of a chemistry experiment.^80^

## THE RESEARCH LIFECYCLE

All scientific investigations generate a much wider range of material than just the results obtained, whether they are numbers or recorded observations. If such investigations are to benefit the wider science community, care is needed in the capture, preservation, and description of all of the material. Equal care is required in recording the subsequent stages of analysis and dissemination. This section examines how Semantic Web technologies can assist the cheminformatics community to achieve what the authors of this review refer to as *continuous curation*, throughout the research lifecycle.

Borkum et al.^80^ highlight the need for ‘collaboration between chemistry scholars and computer and information scientists to develop and deploy the infrastructure, services, and applications that are necessary to enable new models for research and dissemination of the scholarly results of chemistry research’. Frey^15^ identifies three main phases in the research lifecycle: planning, execution, and dissemination. He contends that Semantic Web technology can speed up the planning phase by enhancing the discovery process, not only of relevant information, including publications, but also of people with similar interests and required skills. The e-Science community has encouraged the necessary collaboration by forming virtual organizations, but support for formal virtual organizations (VOs) has waned in favor of groups set up around social networking tools such a LinkedIn, FaceBook, and Google circles.

The execution phase involves the capture of both data and observations in context and, importantly, the curation of that information. Chin and Lansing^106^ set out the basic principles of capture in context, albeit for a biosciences collaboratory but one developed from the CMCS.^65^ They note that context is both physical and scientific and is captured as metadata. They also discuss the importance of data provenance for tracing the evolution of datasets, to which contextual information can also be relevant. To apply these principles in an environment that exploits semantics, it is important to capture information in machine-processable formats. Frey^19^ argues for curation to be an indispensable part of the experimental process, to be designed into every experiment: curation at source. The UK has established a national organization, the Digital Curation Centre, for tackling the challenges of preserving and managing research data.^107^

The ELN is now essential to good practice in capture and curation. ‘ELN and the Paperless Lab’ is a selective compilation of articles written about ELNs in recent years.^108^ This eBook provides a broad range of insights into the evolution of ELNs and the motivations of the experimenters who use them. Previously, Taylor^33^ had reviewed the use of ELNs specifically for chemistry and biology: at that time (2006) he predicted that increased adoption would depend on the technology becoming proven and affordable. More recently, Quinnell et al.^109^,^110^ have reported trials of an ELN with selected undergraduate and postgraduate chemistry students at the University of New South Wales, Australia.

The dissemination phase is, in a sense, recursive, in that collaboration pervades the research lifecycle. Williams reviewed the use of Internet-based tools, including Semantic Web tools, for drug discovery,^57^ concluding that, for commercial organizations, blogs and wikis are more likely to be adopted internally than for external collaboration. Academic institutions are likely to be significantly less inhibited. However, it might be necessary to distinguish between the informal sharing of ideas and the more formal exchange of structured information. Several authors have commented on the antipathy of chemists toward data sharing. In 2008, Downing et al.^111^ conducted a survey of all research chemists at both Cambridge and Imperial College to determine data preservation practices and needs. They found a tendency to store data as hard copy, and where data was preserved electronically, a range of formats were in use. The attitude to storing data in an open repository depended in part on a reluctance to make data available prior to publication, allowing only other group members to see information before publication.

For scientists, publication is the ultimate form of dissemination, so researchers with an interest in semantic and Web 2.0 technologies have been drawn toward approaches that go beyond the traditional paper publishing. Marking up text with a language that conforms to a publicly known schema is one approach, leading Murray-Rust and Rzepa^112^ to propose CML for this purpose. At the same time, Frey et al.^113^ presented a case for *publication at source*, using Grid technology to disseminate information about the conduct of experiments as well as the resulting data: Figure 1 in their paper is an early depiction of the linked data concept.

Shotton^114^ has reviewed progress toward semantic publishing, in which he cites journals published by the Royal Society of Chemistry and particularly the RSC Project Prospect as an exemplar of semantic publishing. The RSC has made significant advances in this area, with RSC Semantic publishing^115^ (as Project Prospect is now known), which is linked to the RSC ChemSpider database.^116^ Manuscripts submitted to the RSC are annotated with semantic markup to highlight the important chemical data, particularly the structures. The data markup includes links to the relevant text and additional property data. Subsequently, search engines can exploit the annotations, for instance to discover papers that relate to a particular structure. The approach taken by this RSC project demonstrates the advantages of publication in a format that is compatible with Semantic Web technologies, which can in turn generate further insights from such semantically enriched information. RDF functionality has recently been added to the ChemSpider interface, enabling Richard Kidd, Informatics Manager at the RSC, to blog about what might be possible with semantic chemistry.^117^ Martinsen^118^ refers to the RSC project when discussing semantic tagging in his report on the Evolving Network of Scientific Communication session at the 223rd meeting of the American Chemical Society. His report notes the increasing impact of Web 2.0 technologies, a theme taken up by Bachrach,^93^ as discussed in the Semantic Web Technology section of this review.

## DEPLOYING THE SEMANTIC WEB

The design and discovery of new drugs is the most prominent application of cheminformatics and therefore the natural area for deploying Semantic Web technologies. Willett^2^ identifies structure search and property modeling as two related areas at the foundations of modern cheminformatics. The eMolecules database provides for substructure and molecular similarity searches, but does not currently exploit semantic labelling.^119^ ChemSpider provides equivalent facilities and also provides Web services for querying and accessing its database.^116^ Although ChemSpider is moving toward including semantic methods,^117^ these are not yet evident on its Web site. The CrystalEye database accumulates crystallographic structures, to which it can add semantic markup when converting the data to CML.^120^ Richard et al.^121^ have discussed the value of semantic markup in associating structures with important properties, in their case toxicity data. However, the overall message is that structure search has been notably slow to adopt Semantic Web technology. The issue is potentially quite fundamental in that structure search is mostly about substructure search and efficient algorithms exist for this and it is not clear that this substructure view of the world is actually compatible with the semantics of the whole structure.

Quantitative structure activity relationships (QSAR) are the established basis for deriving structure property relationships that can be used in drug design to predict the chemical properties of new structures. QSAR modelling has made reasonable progress in using Semantic Web technologies, such as RDF: Willighagen et al.^122^ give a number of examples of linking RDF and QSAR modeling; Chepelev and Dupontier^9^ use SADI to link to QSAR functionality in the CDK (Chemistry Development Kit).

As well as investing in the discovery of new drugs, the pharmaceutical industry also devotes resources to finding new uses for known drugs. Oprea et al.^123^ have recently reviewed the techniques used to find new uses. They argue that Semantic Web technologies could contribute to an integrated approach to discovering the associations on which drug-repurposing efforts depend.

The Indiana University School of Informatics has developed a variety of tools that deploy the Semantic Web for drug discovery. The best known is arguably Chem2Bio2RDF,^29^ but Wild^124^ describes the full range of tools on his home page. WENDI looks particularly interesting in that it uses an RDF inference engine to reveal potential but not otherwise obvious biological applications for chemical compounds.^125^

### Workflows, Web Services, and Interoperability

The authors have recently reviewed the deployment of workflows and Web services for drug design and discovery^22^ and concluded that the increasing use of Web services means that it is becoming easier to use workflows and workflow systems to provide assemblies of services that are useful in drug design and discovery. Kuhn et al.^126^ have developed CDK-Taverna to provide a workflow engine specifically for cheminformatics by developing a Taverna plugin to integrate CDK: in their article, they provide six scenarios as examples of the use of CDK-Taverna. ‘Web 2.0 for Grids and e-Science’ is the subject of a book chapter by Fox et al.^127^ Previously, Curcin et al.^24^ had paid particular attention to the role semantics in their review of Web services for the life sciences.

Although workflows can use Semantic Web technologies to communicate the characteristics of data in precise manner, cheminformatics applications have to maintain that precision when interfacing with semantic methods. Willighagen et al.^122^ examine the interoperation of a range of molecular chemometrics applications and conclude that these techniques can integrate successfully with RDF data. The OpenTox project^128^ aims to provide semantic services to assist integration of toxicology information with the rest of the drug discovery process. The Chem2Bio2RDF repository exploits semantics to facilitate interoperation between chemistry and biology by integrating chemogenomics repositories with other chemical biology resources.^42^ In the context of managing research projects, Alsberg and Clare^32^ demonstrate the use of MediaWiki for handling the interoperation of the various aspects of chemometric research projects. However, among the shortcomings that they point out are the lack of semantic annotation and an outstanding issue with integrating large amounts of structured data: clearly there is scope for introducing further semantic technology.

### Open Data

The activities of the Linking Open Drug Data task force^77^ were covered in the Linked data section of this review. The Open PHACTS consortium aims to develop an open source, open standards, and open access platform as the basis of an open pharmacological space (OPS).^47^ The consortium will use trusted third parties to resolve security issues related to proprietary data. Hohman et al.^129^ foresee open access, open source, and open collaboration as the future for drug discovery. They argue that a growing community of networked scientists, sharing data and expertise, can achieve more efficient discovery of new candidate drug molecules. However, if their vision is to be realized, collaborating researchers will need to be sure of the semantics of the data they access ‘out in the open’.

The ChemCloud infrastructure, discussed above, is based on linked open data principles.^78^ The Blue Obelisk movement^86^ was founded specifically to promote open source, open standards, and open data: the members of the group continue to do so.^102^ Jean-Claude Bradley is a leading exponent of open science: he provides all the experimental results from his work on antimalarial compounds online.^130^ Neylon and Todd have also made some of their laboratory notebooks available and in the latter case a whole research project is coordinated in public view as Project Lab Books on the ourexperiment.org site; for example, the Pictet–Spengler route to Praziquantel.^131^

Todor^132^ surveys a range of use cases in his presentation: ‘Semantic Linked Data Integration for Chemical eScience’. Hunter et al.^133^ have focused on the annotation of 3D crystallographic models, essentially a form of curation. The main tool they use for their AnnoCryst system is Annotea, which is a W3C Semantic Web project that uses RDF schema.^134^ Adams and Murray-Rust^135^ published an early example of deploying semantic technologies for a specific application, polymer informatics, in 2008.

## CONCLUSION

Rajarshi Guha's blog^136^ illustrates that applications of Semantic Web technologies in cheminformatics are still the subject of active discussion. It has become clear that the role of the Semantic Web in promoting systematic use of agreed metadata for integration of data is currently the most powerful driving force in the development of Semantic Web tools. The possibilities for reasoning over the semantically rich data produced are still in their infancy. The major advances that have been made in the Chemical Semantic Web in the last few years have brought chemical informatics into closer alignment and integration with bioinformatics. The RDF description works best in an ‘open world’ both in the technical and administrative meaning of the word. Developments have been faster where data was easily available, but other routes to accessing the necessary data are increasing possible and will ensure that the exciting demonstration based on freely available data can spread to environments were the data is necessarily more controlled and restricted.
